# Influence of Compaction Methods on Properties of Roller-Compacted Concrete Pavement Wearing Surfaces

**DOI:** 10.3390/ma18030492

**Published:** 2025-01-22

**Authors:** Justyna Stępień, Anna Chomicz-Kowalska, Magdalena Tutaj-Dudała, Michał Dudała, Krzysztof Maciejewski, Piotr Ramiączek, Mateusz Marek Iwański

**Affiliations:** 1 Department of Transportation Engineering, Faculty of Civil Engineering and Architecture, Kielce University of Technology, Al. Tysiąclecia Państwa Polskiego 7, 25-314 Kielce, Poland; justynas@tu.kielce.pl (J.S.); kmaciejewski@tu.kielce.pl (K.M.); piotrr@tu.kielce.pl (P.R.); 2 General Directorate for National Roads and Motorways (GDDKiA), ul. Paderewskiego 43/45, 25-950 Kielce, Poland; mtutaj@gddkia.gov.pl; 3 Przedsiębiorstwo Robót Inżynieryjnych “Fart” Sp. z o.o., 25-116 Kielce, Poland; laboratorium@fartkielce.pl; 4 Department of Building Engineering Technologies and Organization, Faculty of Civil Engineering and Architecture, Kielce University of Technology, 25-314 Kielce, Poland; matiwanski@tu.kielce.pl

**Keywords:** roller-compacted concrete, RCC, compressive strength, concrete density, compaction, design of rolled concrete mixtures

## Abstract

The present study investigates the effects of different compaction methods on the properties of roller-compacted concrete (RCC) used for road pavements. The study focuses on comparing the Proctor compaction method utilizing different compaction efforts and molds (2.5 kg rammer with three layers of 56 blows and 4.5 kg with three and five layers of 56 blows, cylindrical and cube molds) with a slab compactor in static and vibratory setting. The samples produced in a slab compactor were obtained by drilling from the prepared slab. The evaluated properties of the samples included compressive strength and bulk density. The study involved a C25/30 concrete with the intention to be used in low volume roads according to national standards. The study concluded that the utilization of Proctor compaction and slab compactor with vibratory setting provided similar levels of strength performance of the RCC mixture, regardless of the shape of the Proctor compacted samples. In terms of the bulk densities, the main differentiating factor in the case of Proctor compaction was the weight of the rammer. The compressive strength of the samples was also strongly related to their bulk densities.

## 1. Introduction

Various pavement technologies are used in road construction [1,2]. Currently, concrete technology in road construction is used most frequently to pave concrete wearing courses on roads with heavy traffic on motorways and highways [3,4]. These roads are mostly made as jointed plain concrete pavement or concrete pavement with continuous reinforcement with poured concrete. Undoubtedly, this increases the cost of investment and makes it necessary to use specialized equipment. For lower-category roads, the use of this technology is often seen as too expensive and problematic, one of the reasons being the impossibility of introducing traffic to the pavement quickly after paving. Rolled compacted concrete combines the advantages of asphalt and concrete technology. The construction of pavements using this technology can be carried out with the use of asphalt paving machinery, reducing the entry cost for this technology. Light traffic may be introduced on these pavements in a very short time after the installation of the rolling concrete wearing course. Therefore, it is an interesting alternative to asphalt and poured concrete pavements for municipal and district roads, where the low construction costs and the low road operating costs are favorable.

Roller-compacted concrete (RCC) technology has become popular in North America. Canada and the United States are the world leaders in the number of roads made using this technology. In Europe, sections of roads with a surface made of rolled concrete can be found in Western EU countries. These are mainly local roads but also parking and maneuvering areas of airports and port quays, as well as parking lots. In Asia, the technology of roller-compacted concrete has been used in the construction of large hydrotechnical structures (dams) [5,6,7]. As a trend observed worldwide, many countries have shown interest in implementing RCC pavements. Among these, heavy and light industrial areas, ports, intermodal facilities [8], airport service areas, arterial streets, local streets, road widening or over-widening, berms [4], and storage yards have been highlighted as the most adequate for using RCC. In the United States, RCC is used to build parking lots and maneuvering areas. The US military commonly uses rolled concrete technologies to pave large areas.

Road construction based on rolled concrete technology is an interesting alternative to bituminous technology. With comparable construction costs, a high pace of work, and low operating costs, it may gain more popularity in the near future.

Surfaces in the rolled concrete technology are characterized by the following:

- Use of standard machines for asphalt paving (pavers, smooth steel, and tire rollers);
- The ability to be subjected to traffic loads much faster than in the case of classic concrete technology with the use of poured concrete, even 48 h after paving;
- Speed of the construction process—renovation including removal of the old surface takes up to 7 days and the speed of laying the new surface ranges from 60 to 120 m per hour;
- High availability of concrete plants.

Compared to flexible pavements, the biggest advantage of RCC is the lack of its susceptibility to permanent deformation. Due to this, the rolled concrete technology can be used in places where heavy vehicle slow traffic is encountered (intersection shields, roundabouts, bus lanes, and slow traffic lanes). The concept of rolled concrete assumes that it is a concrete mixture with aggregate composition and moisture content selected in such a way that it has properties similar to the soil at an optimum moisture content prior to the setting of the hydraulic binder, allowing maximum compaction by rolling. The RCC mixtures for pavements offer different advantages, such as being a more sustainable alternative to traditional concrete pavements, as they require less cement [9] and can incorporate recycled materials [10]. RCC pavement comprises a special type of concrete mixture, which is widely popular due to its economic and fast construction characteristics [10,11,12]. Road surfaces made of RCC are made with the use of typical asphalt pavers and compacted with vibratory rollers weighing more than 10 t [13].

The adaptation of RCC to the climatic conditions of Central and Eastern European countries requires such a design of mixtures that they meet the requirements of compressive strength and frost resistance in the presence of deicing agents (peeling). The impact of selected service conditions (relative humidity, temperature) and various mix designs (different cement, water, and additive contents) on the RCC performance, focusing on the shrinkage phenomenon and resistance to cracking, should be thoroughly investigated in the future [14,15,16,17]. One of the most significant properties of a paved layer of RCC is its compaction, evaluated by the ratio of the bulk density of the paved material to its maximum bulk density, typically obtained using the modified Proctor method. Generally, RCC should be compacted at least to 98% of Proctor bulk density (wet) to ensure adequate freeze–thaw performance and strength [18,19]. In this sense, it is important to be able to perform adequate quality control tests to determine the correct compaction of the road surface. The formation of laboratory specimens should lead to their proper compaction.

The basic methods for the preparation and curing of specimens intended for concrete strength tests are described in the EN 12390-2 standard [20]. Adequate curing and compaction of cementitiously bound materials are fundamental for their performance [21,22]. The compaction of the specimens should be carried out in a minimum of two layers. Each layer should not be thicker than 100 mm. After laying the concrete in the mold, its compaction must be started immediately and carried out in such a way as to prevent segregation of the components. It is recommended to not allow the laitance to precipitate on the surface during compaction [20,23]. The above methods of compacting specimens are difficult to implement in the case of concretes with a moist consistency (K1 consistency according to the Vebe method) characterizing rolled concrete mixtures. Compaction using the above methods may lead to insufficient compaction of the specimens, which results in obtaining inadequate strength parameters. This was shown in a number of studies where the required compaction level was difficult to achieve using the vibrating table method [24,25,26].

In the case of RCC mixtures, the method of compacting specimens described in the EN 13286-50 standard [23] seems to be more appropriate. This standard describes methods for producing specimens from hydraulically bound mixtures with which a Proctor apparatus or a vibrating table is used for compaction. Compaction is continued until the required density is reached [23]. It was shown that the modified Proctor method used in the compaction of RCC samples adequately represents the field strength performance of such mixtures, however, the high impact energy used in the Proctor method may lead to the breaking of aggregates and therefore change the structure of the mixture [24].

Additionally, a gyratory compactor was considered in a number of studies as it reflects the shifting of the aggregates under the passing roller as well as incorporating the vertical pressure required for the compaction of RCC mixtures [25,27]. To simulate the operation of a road roller for compacting RCC specimens in the laboratory, the use of an asphalt slab compactor could also be considered, but to date, there are no clear technical guidelines for its use in RCC technology.

There is ongoing work evaluating different compaction methods of RCC mixtures in attempts to adequately reflect their field performance. Based on the current understanding of the topic, the present paper investigates the effects of modifications to the Proctor compaction method and, for the first time, the utilization of a slab compactor in the preparation of roller-compacted concrete mixes is evaluated. For this reason, this paper investigates the influence of RCC compaction methods on their basic properties (compressive strength, density, compaction) in order to determine differences between the different methods.

## 2. Materials and Methods

### 2.1. Materials

**Figure 1:**
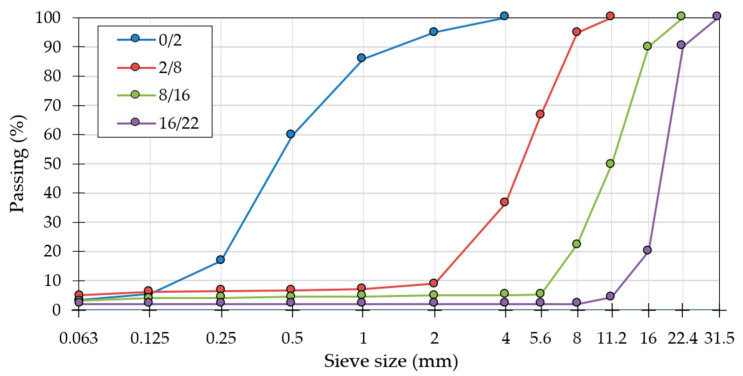
Particle size distribution of aggregates used in the RCC mineral mixture.

The design of the mixture included only natural aggregates. The composition of the RCC mixture was based on the following:

- Portland-composite cement with fly ash CEM II/B-V in accordance with the EN 197-1 standard [28] about standard strength class 32.5R in accordance with the EN 196-1 standard [29];
- Aggregates with particle sizes of 0/2, 2/8, 8/16, an 16/22.4 mm in accordance with the EN 12620+A1 standard [30] (Figure 1, Table 1 and Table 2);
- Mixing water for concrete in accordance with the EN 1008 standard [31].

The results of the aggregate sieve analysis are presented in Figure 1.

Fine and coarse aggregates were obtained from mines located in Poland (Świętokrzyskie Voivodeship). Natural sand (yellow, washed) with the properties given in Table 1 was used as fine aggregate. The coarse aggregate was limestone, and the following chemical composition was obtained from the producer (mine):

- SiO_2_—4.18%;
- CaO—52.30%;
- MgO—0.42%;
- Fe_2_O_3_—1.28%;
- Al_2_O_3_—0.25%.

Table 2 presents the geometric and physical properties of the coarse aggregates used in the mineral mixture of RCC.

For the designed mixture, the strength class C25/30 of concrete according to the EN 206 standard [32] was assumed as recommended for wearing layer for the light traffic (<500,000 ESAL_100kN_), which is the subject of this study.

**Table 1:** Basic properties of fine aggregates used in the RCC mineral mixture.

Property	Test Method	Performance Properties/Category
Aggregate sizes (d/D)	EN 933-1 [33]	0/2
Grading	EN 933-1 [33]	G_F_85
Fines content	EN 933-1 [33]	f_3_
Apparent particle density ρ_a_, Mg/m^3^	EN 1097-6 [34]	1.58 ± 0.10
Total sulfur	EN 1744-1 [35]	≤1.0

**Table 2:** Geometric and physical properties of the coarse aggregates used in the RCC mineral mixture.

Property	Test Method	Performance Properties/Category		
Aggregate sizes (d/D)	EN 933-1 [33]	2/8	8/16	16/22.4
Grading	EN 933-1 [33]	G_C_90/15	G_C_90/15	G_C_90/15
Tolerance of grading	EN 933-1 [33]	G20/17.5	G20/15	G20/15
Flakiness index	EN 933-3 [36]	Fl_15_	Fl_15_	Fl_10_
Grain density:	EN 1097-6 [34]			
Apparent particle density ρ_a_, Mg/m^3^		2.674	2.684	2.681
Oven-dried particle density ρ_rd_, Mg/m^3^		2.646	2.656	2.652
Saturated and surface-dried particle density ρ_ssd_, Mg/m^3^		2.656	2.666	2.663
Fines content	EN 933-1 [33]	f_2_	f_2_	f_2_
Resistance to fragmentation, Los Angeles test method	EN 1097-2 [37]	LA_25_	LA_25_	LA_25_
Freeze–thaw resistance	EN 1367-1 [38]	F_1_	F_1_	F_1_
Resistance to polishing	EN 1097-8 [39]	PSV_44_	PSV_44_	PSV_44_
Loose bulk density ρ_o_, Mg/m^3^	EN 1097-3 [40]	1.628	1.708	1.680

The properties of the aggregates indicate their suitability for RCC in accordance with the EN 12620+A1 standard [30]. They are characterized by appropriate continuous grain distribution (Figure 1) and geometric and physical properties (Table 1 and Table 2) that allow one to achieve the desired characteristics of the RCC concrete mix.

### 2.2. Methods

#### 2.2.1. Experimental Methodology

The study involved evaluation of the state-of-the-art in the subject area, selection and evaluation of the input materials, and design of the RCC concrete mixture, which included assessment of the optimum moisture content in the mix using the Proctor method, manufacturing of the mixtures and compacting the samples, sample curing, and experimental testing.

The experimental tests included the evaluation of the influence of the compaction method using the Proctor rammer (as in EN 13286-50 standard [23]) and the slab-forming compactor (as in EN 12697-33 standard [41]) with a static and vibratory setting on the following properties of the RCC intended for the wearing course of the road surface:

- Compressive strength after 7 and 28 days (MPa) in accordance with the EN 12390-3 standard [42];
- Bulk density (g/cm^3^) on 28-day strength test specimens with the use of the geometric method for cube and cylindrical specimens, and with a sand volume meter for specimens drilled in the slab in accordance to the ASTM D 1556 [43];
- Percent bulk density (%) in relation to the modified Proctor method.

#### 2.2.2. Methods for the Design of the RCC Mixture

According to US guidelines, selecting the optimal composition for RCC is usually based on experimental methods. The two most popular methods are the method published by the US Army Corps of Engineers (USACE) [44] and the method published by the American Concrete Institute (ACI) [45]. The main goal of these methods is to create a well-compacting mix, which is stable during compaction, resulting in durable concrete with minimal cement consumption.

On the basis of the aggregate grain size curves, the composition of the RCC mixture was developed. It was decided to follow a design method using the limit grading curves method of the American Concrete Institute [45].

The modified Proctor method (EN 13286-50 standard [23]) was used to determine the moisture content of the optimal RCC mixture and its dry density. The dimensions of the Proctor molds and the rammer parameters required according to the EN 13286-2 standard [46] are given in Table 3 and Table 4. For this purpose, a B rammer weighing 4.5 kg and a B mold with internal dimensions of 120 mm in height and 150 mm in diameter were used. Compaction was carried out in 5 layers with 56 blows of the rammer per layer. This allowed the optimum moisture content in the RCC mixture to be determined.

#### 2.2.3. Methods for Preparing the RCC Mixture for Testing

The preparation of the RCC mixture in the laboratory was carried out with the use of dried aggregates, thanks to which it was possible to precisely measure the dosed water. The mixture was produced using a DIEM mixer (Form+Test Prufsysteme, Riedlingen, Germany). In the beginning, coarse aggregates were dosed, and then cement and sand followed. Mixing the dry ingredients took about 60 s. Water was added to the mixture, and the final mixing took about 90 s.

#### 2.2.4. Methods for Producing RCC Test Specimens

**Figure 2:**
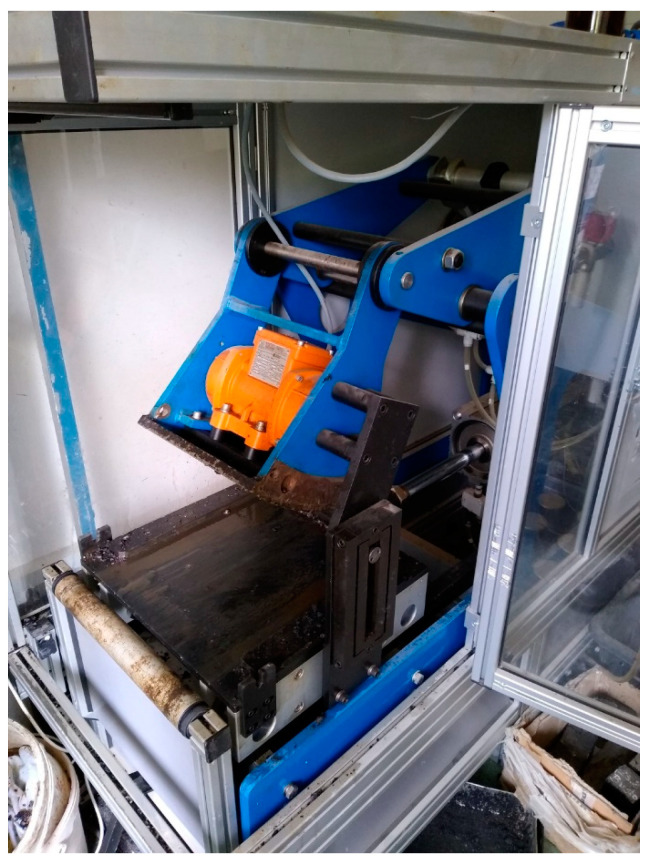
A slab compactor used to produce the 100 mm RCC slabs in the study.

The Proctor method described in the EN 13286-50 standard [23] is typically considered the appropriate method for the preparation and compaction of RCC specimens. For compressive strength tests, cube specimens 150 mm inside (Figure 2) and specimens in cylindrical molds with a diameter of 160 and height of 160 mm were prepared and compacted using the A and B Proctor rammer as follows:

- Proctor rammer A (2.5 kg), compaction in 3 layers with 56 blows per layer;
- Proctor rammer B (4.5 kg), compaction in 3 layers with 56 blows per layer;
- Proctor rammer B (4.5 kg), compaction in 5 layers with 56 blows per layer.

The second group of RCC specimens was prepared using a slab-forming compactor typically used for producing samples to test the resistance to permanent deformation of asphalt mixtures (Figure 2). Molds with dimensions of 305 × 305 × 100 mm were used and compaction was carried out in two variants:

- In the first variant, six static passes were used for compaction, simulating the operation of a heavy roller;
- In the second, the first two passes used static pressing force, while the next four included vibration.

The specimens were obtained by drilling the prepared slab to produce samples 100 mm in diameter and 100 mm in height.

#### 2.2.5. Compressive Strength and Density Tests

**Figure 3:**
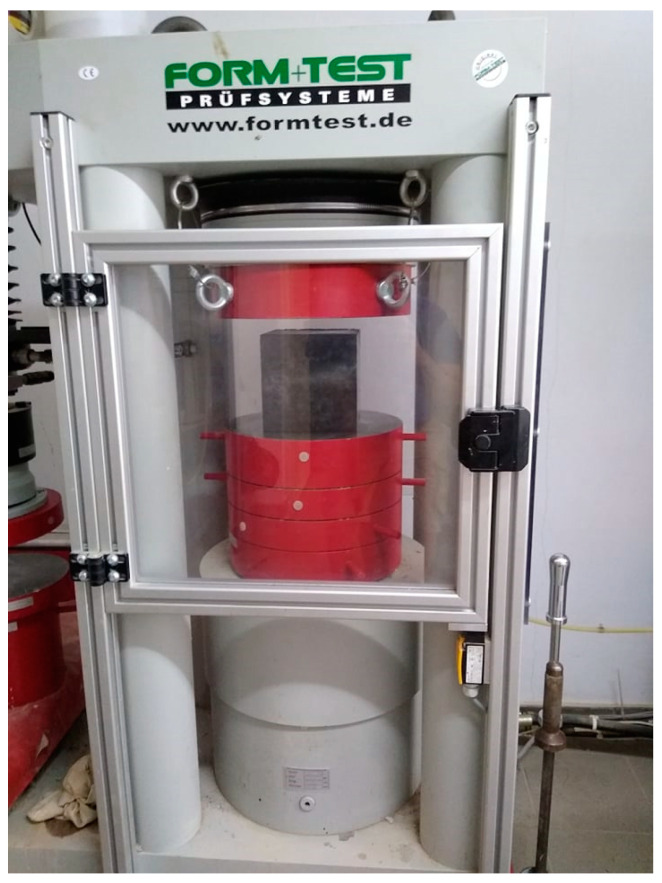
The hydraulic press is used for compressive strength testing of RCC specimens.

Compressive strength tests were carried out for 7-day and 28-day concrete in accordance with the EN 12390-3 standard [42] using a hydraulic press (Form Test Prufsysteme, Riedlingen, Germany) as shown in Figure 3.

The density evaluation for cubic and cylindrical specimens was carried out using the geometric method.

After compaction, the specimens remained in the molds for 24 h. The next day, they were demolded and placed in water at 20 °C (±2 °C) until the day of the test.

## 3. Results

### 3.1. Roller-Compacted Concrete Mix Design

**Figure 4:**
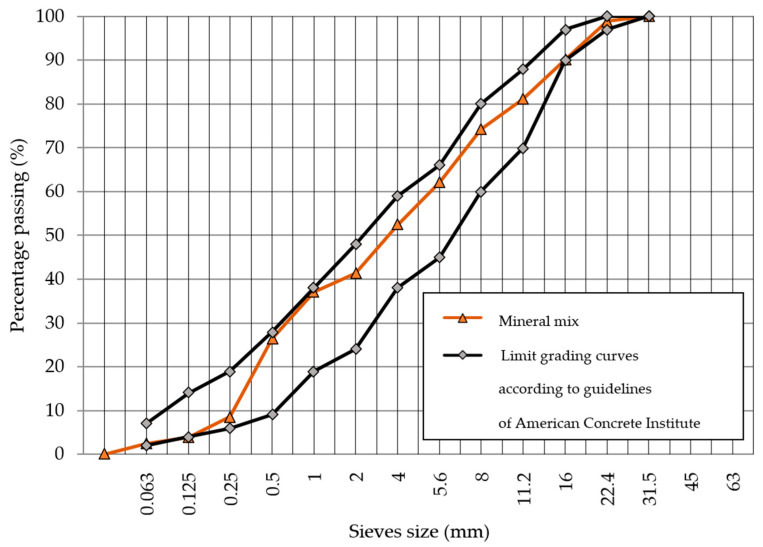
Particle size distribution of mineral mix for RCC with limit grading curves according to guidelines of ACI [45].

**Figure 5:**
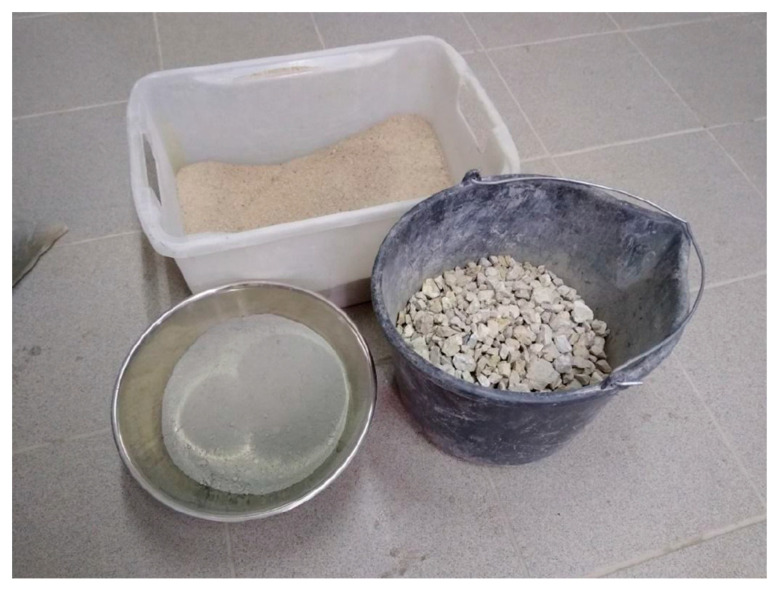
Dry materials used to prepare the RCC mixtures: cement and fine and coarse aggregates.

The granular composition of the RCC mineral mix was determined using limit grading curves according to the guidelines of the American Concrete Institute [45]. Its particle size distribution is presented in Figure 4 and the dry materials used to produce the mix are presented in Figure 5.

The binder content used in this study was assumed to be 280 kg/m^3^ based on the ranges investigated in other studies on RCC [9,47]. This value corresponded to ca. 12.5% of the dry mix weight, which was found to be in the lower bound of the 10–17% range recommended by the ACI [45].

The determination of the optimal moisture content of the optimal mixture and its volume density was prepared using the modified Proctor method [23,48] and is shown in Table 5.

**Figure 6:**
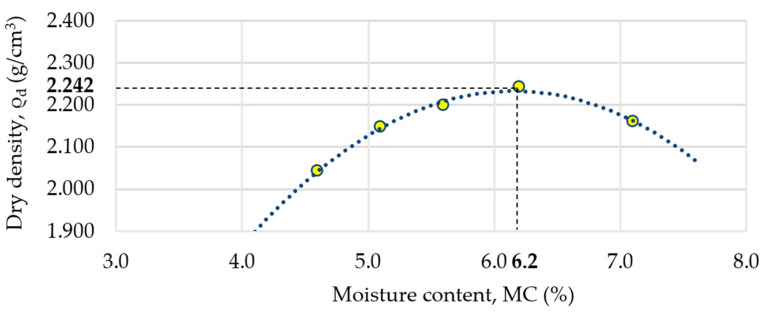
Relationship between moisture content (MC) and dry density (ρ_d_) of the mixture.

Based on the results from Table 5, the optimal moisture content (OMC) was determined at 6.2%, resulting in a maximum dry density of 2.242 g/cm^3^. According to the results obtained, the mixture should contain 148 kg/m^3^ of water to allow its optimum compaction. The relationship between dry density and the moisture content of the mixture is shown in Figure 6.

Finally, the total composition of the concrete mix was determined in Table 6.

### 3.2. Compressive Strength and Bulk Density Tests

**Figure 7:**
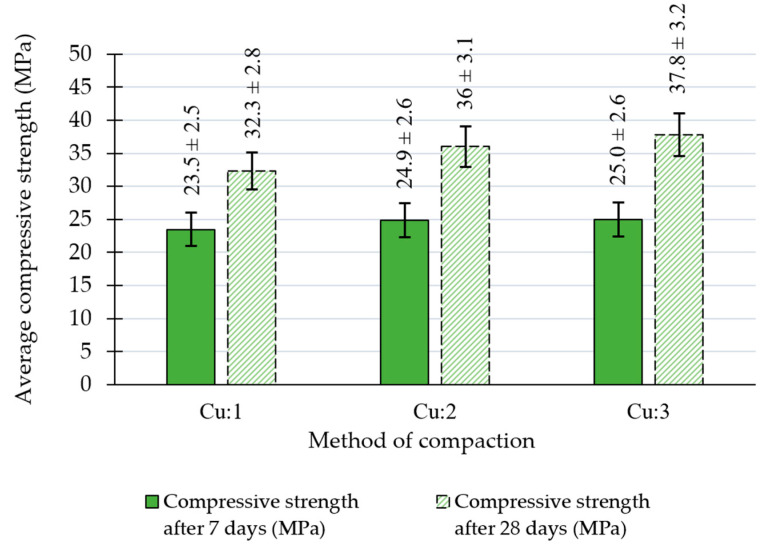
Average compressive strength values after 7 and 28 days determined on Proctor cubic specimens of RCC.

The results obtained on cylindrical samples prepared using different Proctor compaction methods (Cu:1, Cu:2, Cu:3) are shown in Figure 7 and Table 7. The presented bulk density values correspond to the 28-day cured specimens. Based on the results, it can be seen that the largest difference in the density of compacted samples was a result of increasing the rammer weight from 2.5 kg to 4.5 kg, while the number of layers used in the compaction using the heavier rammer had a smaller effect. Along with the difference in the density of the material, the compressive strengths were visibly increased with the Cu:2 and Cu:3 compaction methods employed; in particular, the 28-day strengths were affected more significantly. The percentage increase in the average compressive strength between the 7-day and 28-day cured samples appeared to increase as the compaction effort was increased. This result was obtained because the 7-day compressive strengths varied only by 1.5 MPa while after the 28-day curing, the different compaction methods yielded up to a 5.5 MPa difference in this parameter.

**Figure 8:**
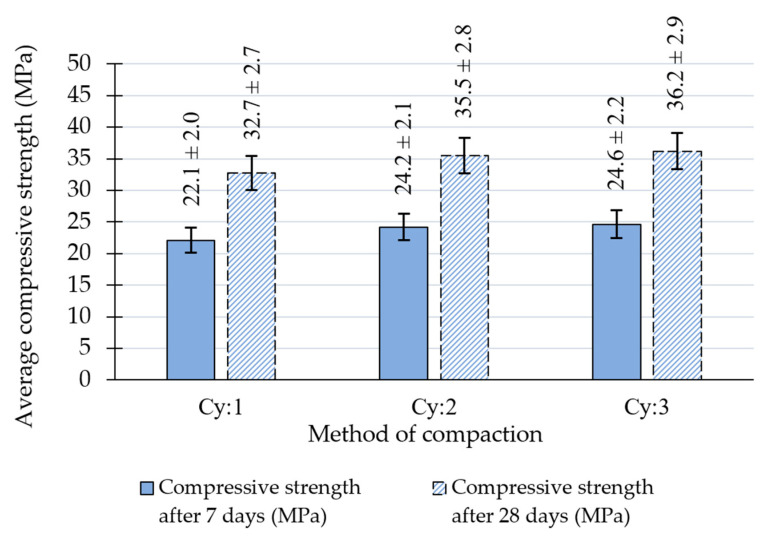
Average compressive strength values after 7 and 28 days were determined on RCC Proctor cylindrical specimens.

Figure 8 and Table 8 show the results obtained on cylindrical Proctor specimens of (Cy:1, Cy:2, Cy:3). The effects of different Proctor compaction efforts in cylindrical specimens yielded similar results as in cubic specimens with the greatest differences in achieved density and compressive strengths between the Cy:1 and the Cy:2 methods and highest values of both parameters obtained using the Cy:3 compaction method. Compared to cubic specimens, cylindrical samples in all cases obtained higher bulk densities, which can be attributed to their smaller volume (approximately 5% difference) and less effective compaction of cubic specimens with a round tamper. Cylindrical specimens achieved similar compressive strengths to cubic ones, despite the effects of their shape on compressive strength, which can be attributed to their higher compaction. In these cases, a similar percentage increase in the mean compressive strength was found after 28 days compared to the result after 7 days regardless of the compaction method.

**Figure 9:**
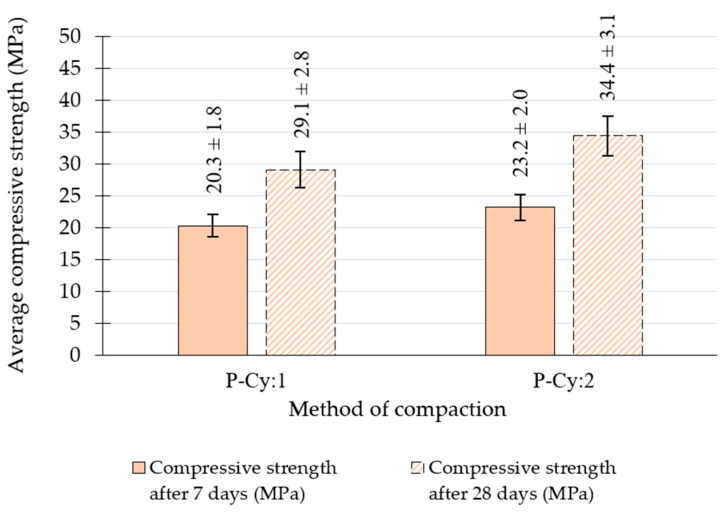
Average compressive strength values determined after 7 and 28 days on specimens drilled from the slab.

Figure 9 and Table 9 present the results of the tests carried out on specimens obtained by drilling from slabs. Four 100 mm in diameter and 100 mm high specimens were drilled from the compacted slabs and tested accordingly. Significant differences in the results obtained with the two variations in this compaction method were attributed to the vibration setting used in the P-Cy:2 method. Significant increases in the density of the compacted specimens and in their 7-day and 28-day compressive strength were recorded when four passes of the vibration-based slab compactor were used. Despite this increase, the samples obtained from the slabs recorded densities and compressive strengths lower than those compacted using the Proctor method. For the P-Cy:2 compaction method, a 5% increase in the average compressive strength after 28 days was obtained compared to the value after 7 days for the P-Cy:1 method.

In order to analyze whether the results obtained meet the requirements of the assumed concrete class, the following conditions (1) and (2) must be met:

(1) $$f_{cm}\geq f_{ck}+4$$

and

(2) $$f_{ci}\geq f_{cm}-4$$

where

- $f_{ck}$—characteristic compressive strength of concrete after 28 days;
- $f_{cm}$—average compressive strength of concrete;
- $f_{ci}$—a single test result of concrete compressive strength determined on a cube specimen.

**Figure 10:**
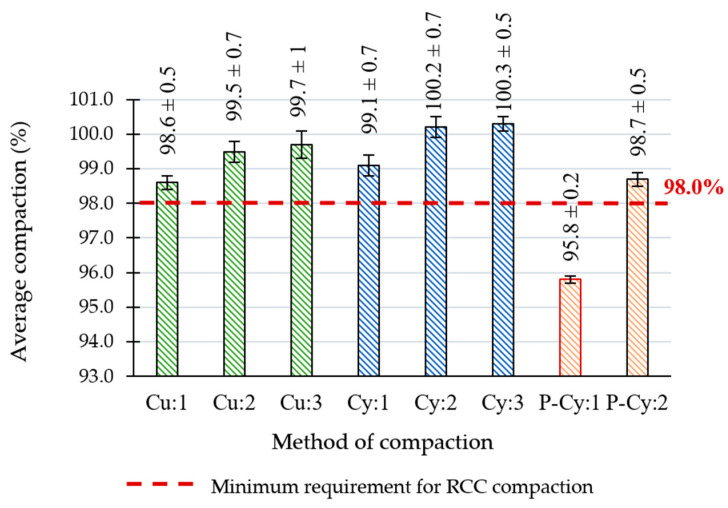
The results of the specimen compaction depend on the compaction method used.

Figure 10 shows the evaluation of the compaction of samples produced using different methods compared to the maximum wet bulk density obtained using the modified Proctor method. The samples drilled from statically compacted slabs were the only ones that did not meet the required 98% compaction rate (as recommended by [18,19], depicted by the dashed line), while those compacted with the use of vibration achieved 98.7% compaction, which was on the lower end of the obtained passing values. It should be noted that the compacted slab had a thickness of only 100 mm, and it is not certain how the compaction would be affected by increasing its thickness. It can also be seen that Proctor-compacted cylindrical specimens obtained highest rates of compaction, again suggesting that the effectiveness of this method was greater in the case of this geometry of specimens.

**Figure 11:**
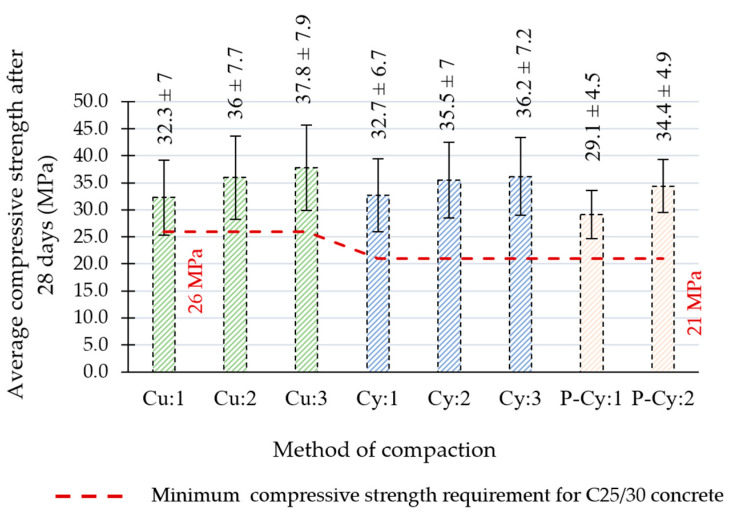
The compressive strength results depending on the method of compaction.

Figure 11 shows the results of the 28-day compressive strengths together with the strength requirements to meet the C25/30 specification respective to the shape of the samples (dashed line). It can be concluded that all specimens met the strength requirements for C25/30 concrete regardless of the compaction method. It can also be seen that when compared to the minimum required strength, the cylindrical specimens had a relatively larger surplus of strength than cubic ones, which can be attributed to their more effective compaction.

### 3.3. Statistical Analysis

The effects of applying different compaction methods were evaluated in terms of 28-day compressive strength and the bulk density of the produced samples using univariate analysis of variance. Table 10 presents the estimates of the effects associated with the evaluated compaction methods and corresponding p-values.

**Figure 12:**
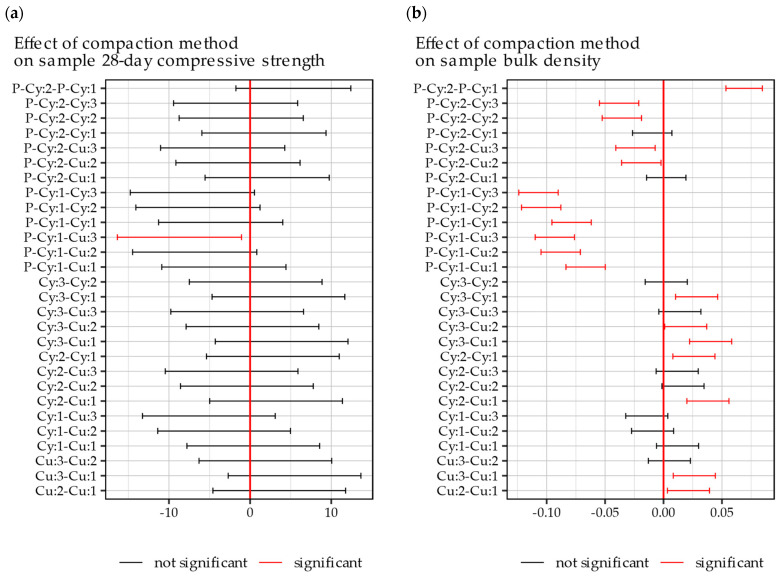
Results of post-hoc Tukey HSD multiple comparison tests for (**a**) 28-day compressive strength and (**b**) bulk density.

Based on the presented analysis, it can be seen that the different compaction methods had greater effects on the bulk density of the produced samples than on their compressive strength. Only the Cu:3 method produced samples significantly (*p* < 0.05) differing in compressive strength among those evaluated. In the case of the bulk density, however, the effects of utilizing different compaction methods were significant in six out of seven cases. Based on the significance of the mentioned effects, a post-hoc analysis using the Tukey HSD test was conducted. The results of Tukey multiple comparison tests are presented in Figure 12 for evaluating the compaction methods in pairs.

The presented figures allow for assessing whether the measured differences in the evaluated pairs of compaction methods were statistically significant (p < 0.05). As shown in Figure 12a and reflecting the results of the analysis of variance, only the Cu:3 and P- Cy:1 pair of sample preparation methods yielded significantly differing results in terms of the 28-day compressive strength. That is, only between the methods that resulted in the highest and the lowest compressive strength was this difference statistically significant. Regarding the results of the bulk density, a number of observations should be noted. First, in the Proctor-compacted samples in both cubic and cylindrical samples, method 1 differed significantly from methods 2 and 3, and there was no significant difference between methods 2 and 3 (i.e., Cu:2 and Cu:3 as well as between Cy:2 and Cy:3). Second, when the weight of rammer, number of layers, and number of blows were equal, similar bulk densities were obtained in samples with both cubic and cylindrical molds. Third, only the slab compactor method utilizing vibratory action achieved bulk densities similar to those obtained in Proctor-compacted specimens (Cu:1 and Cy:1).

Based on the engineering knowledge, literature review, and analysis of Figure 12a,b, it was ascertained that a relationship between sample density and compressive strength should be measurable. A Pearson correlation coefficient between the 28-day compressive strength and bulk density of the evaluated samples was calculated with a relatively high result of R = 0.746. The linear relationship between these parameters was quantified to

28 day compressive strength [MPa] = 78.68 ∙ bulk density [g/cm^3^] − 151.16 (3)

**Figure 13:**
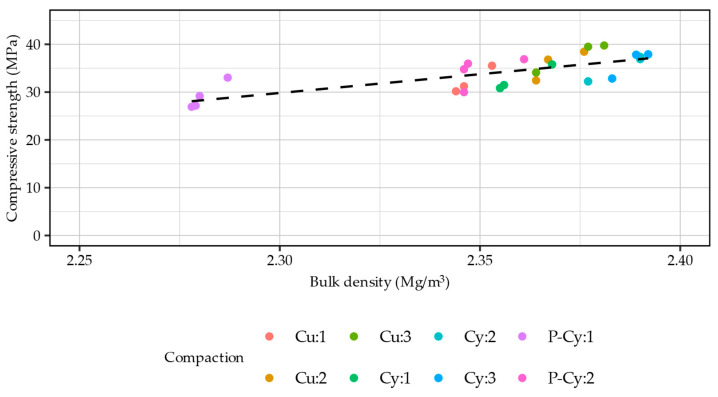
Relationship between the 28-day compressive strength and bulk density of the investigated RCC samples.

The relationship between the bulk density of the evaluated samples and their 28-day compressive strength is presented in Figure 13. The relationship was highly linear, despite the gap between the densities of the samples produced using P-Cy:1 and other methods. The regression model was characterized by a coefficient of determination of R^2^ = 0.538, with both coefficients and the model itself being statistically significant (*p* < 0.05).

## 4. Discussion

In the case of roller-compacted concrete, an adequate level of compaction is one of the critical parameters that has to be achieved both in the field and in the laboratory to adequately represent its properties. Compared to typical construction concretes, this characteristic distinguishes the design of RCC. Obtaining the required 98% density of the wet Proctor bulk density in compacted RCC samples can be most easily obtained in sample fabrication by utilizing the same Proctor compaction method, which can be modified in terms of the compaction effort by using different molds, rammers, material layering, and number of blows. Given the fact that rollers are used in the field to compact the RCC mixture, the utilization of a slab compactor presents itself also as a feasible laboratory method for producing representative samples.

In the case of the specimens compacted using the heavier 4.5 kg rammer, the number of layers in which the mixture was compacted did not significantly affect the bulk density of the final sample. What is more, the resulting bulk densities of the proctor samples were differentiated only by the weight of the used rammer and not by the type of mold. It was also shown that even the use of a lighter rammer in conjunction with compaction in three layers permits obtaining adequate compaction of the RCC mixture. More preferable, however, is the use of a heavier 4.5 kg rammer with three layers, resulting in nearly identical results as with five layers. These changes could potentially preserve the integrity of aggregates, which was shown by other researchers to be an issue regarding this method [24].

While it is well documented that in general cases the compressive strength of concrete measured in destructive tests is dependent on the shape, dimensions, volume, and aspect ratio of the samples [49,50], the effects of different compaction methods in the present study were in most cases non-significant. Most prominently, the differences in the strength of samples produced in cubic and cylindrical molds were significantly smaller than the differences in the respective strength requirements. The results obtained in the study have shown that despite utilizing different compaction methods, the compressive strength of the RCC material was mostly dictated by the level of compaction. This relationship was linear, even regarding the fact that the P-Cy:1 method (utilizing static slab compactor) failed to provide the required 98% compaction level, which did not result in any additional adverse effects in the compressive testing. It should be noted, however, that frost resistance still could be significantly affected in this case.

## 5. Conclusions

The present study concerned the effects of different compaction methods on the basic properties of rolled compacted concrete intended for the wearing course of the road surface. The most important findings include the following:

- The shape of the Proctor samples (cylindrical, cubic) did not significantly affect the strength of RCC samples;
- Samples produced in the slab compactor with the vibration setting performed similarly to the Proctor samples in terms of compressive strength;
- Different compaction methods produced more significant differences in bulk densities than in the compressive strengths of the samples;
- Comparable bulk densities were obtained in cylindrical and cubic molds when the same Proctor rammer was used;
- Only the slab compactor samples prepared without the use of vibration did not meet the required compaction of 98%.

Based on the obtained results and findings of other studies, the following methods can be proposed for further investigation to alleviate the potentially negative effects of the modified Proctor compaction on the integrity of aggregates in RCC mixtures:

- Decreasing the number of layers in which the material is compacted from 5 to 3;
- Utilizing the 2.5 kg rammer;
- A change in compaction method to the one utilizing a slab compactor with a vibration setting.

Future studies should involve an evaluation of the frost resistance of the produced mixtures and the effects of the compaction methods on air-entrained concrete. Further work should include comparing the laboratory-obtained results with field samples and the effects of compaction methods on the internal structure of the material—including assessment of potential aggregate breaking and segregation.

## Figures and Tables

**Table 3:** Dimensions of new cylindrical test molds in accordance with [46].

Proctor Mould	Diameter (mm)	Height (mm)	Thickness	
			Wall	Base Plate
A	100.0 ± 1.0	120.0 ± 1.0	7.5 ± 0.5	11.0 ± 0.5
B	150.0 ± 1.0	120.0 ± 1.0	9.0 ± 0.5	14.0 ± 0.5
C	250.0 ± 1.0	200.0 ± 1.0	14.0 ± 0.5	20.0 ± 0.5

**Table 4:** Essential requirements of new rammers in accordance with [46].

Rammer	Essential Requirements		
	Mass of Rammer (kg)	Diameter of the Base (mm)	Height of Fall (mm)
A	2.50 ± 0.02	50.0 ± 0.5	305 ± 3
B	4.50 ± 0.04	50.0 ± 0.5	457 ± 3
C	15.00 ± 0.04	125.0 ± 0.5	600 ± 3

**Table 5:** The course of determining the optimal moisture content and the maximum dry density of the mix.

Specimen No. (Markings)	Mold Weight	Mold Weight with Specimen	Mold Volume	Moisture Content	Bulk Density	Dry Density
	(g)	(g)	(mL)	(%)	(g/cm^3^)	(g/cm^3^)
P1	9641.0	14171.9	2120.6	4.6	2.137	2.043
P2	9641.0	14428.4	2120.6	5.1	2.258	2.148
P3	9641.0	14563.6	2120.6	5.6	2.321	2.198
P4	9641.0	14689.2	2120.6	6.2	2.381	2.242
P5	9641.0	14546.5	2120.6	7.1	2.313	2.160

**Table 6:** Composition of the RCC mix.

Ingredients of the Mix	Quantity (kg/m^3^)	Percentage
Natural sand, 0/2 mm	785	32.8
Limestone, 2/8 mm	628	26.3
Limestone, 8/16 mm	354	14.8
Limestone, 16/22.4 mm	196	8.2
CEM II/B-V cement	280	11.7
Water	148	6.2

**Table 7:** Results of the increase in average compressive strength after 28 days relative to the result after 7 days and bulk density determined in Proctor-compacted cube specimens.

Proctor Cubic Specimens 150 mm in Side					
Method of Compaction	Rammer Type	Number of Layers	Number of Blows	Increase in the Average Compressive Strength After 28 Days in Relative to Result After 7 Days	Average Bulk Density
	(kg)	(-)	(-)	(%)	(g/cm^3^)
Tamping (Cu:1)	2.5	3	56	37.4	2.348
Tamping (Cu:2)	4.5	3	56	44.6	2.369
Tamping (Cu:3)	4.5	5	56	51.2	2.374

**Table 8:** Results of the increase in average compressive strength after 28 days relative to the result after 7 days and bulk density determined in Proctor-compacted cylindrical specimens.

Proctor Cylindrical Specimens 160 × 160 mm					
Method of Compaction	Rammer Type	Number of Layers	Number of Blows	Increase in the Average Compressive Strength After 28 Days in Relative to Result After 7 Days	Average Bulk Density
	(kg)	(-)	(-)	(%)	(g/cm^3^)
Tamping (Cy:1)	2.5	3	56	48.0	2.360
Tamping (Cy:2)	4.5	3	56	46.7	2.385
Tamping (Cy:3)	4.5	5	56	47.2	2.388

**Table 9:** Results of compressive strength and bulk density determined in specimens drilled from a slab.

100 × 100 mm Specimens Drilled from the Slab				
Method of Compaction	Number of Static Passes	Number of Passes with Vibration	Increase in the Average Compressive Strength After 28 Days in Relative to Result After 7 Days	Average Bulk Density
	(-)	(-)	(%)	(g/cm^3^)
Slab compactor (P-Cy:1)	6	0	43.3	2.282
Slab compactor (P-Cy:2)	2	4	48.3	2.351

**Table 10:** Analysis of variance table for evaluating the effects of compaction methods on the compressive strength and bulk density of RCC samples.

Independent Variable	28-Day Compressive Strength (MPa)		Bulk Density (Mg/m^3^)	
Effect	Estimate	*p*-Value	Estimate	*p*-Value
Intercept	32.303	<0.001	2.348	<0.001
Cu:2	3.593	0.151	0.021	<0.001
Cu:3	5.477	0.035	0.026	<0.001
Cy:1	0.393	0.872	0.012	0.035
Cy:2	3.197	0.199	0.038	<0.001
Cy:3	3.883	0.123	0.040	<0.001
P-Cy:1	-3.231	0.167	-0.067	<0.001
P-Cy:2	2.099	0.362	0.002	0.642

## Data Availability

The raw data supporting the conclusions of this article will be made available by the authors on request. All of the described findings, analyses, conclusions and discussions in the paper are based on the results directly shown, provided and disclosed in the manuscript in form of tables and figures. These either represent the raw data (when single values were obtained), mean values with confidence intervals (when multiple samples were tested) or statistical analyses performed on raw data. Only the raw datasets were not provided.
